# Designing Second Generation Anti-Alzheimer Compounds as Inhibitors of Human Acetylcholinesterase: Computational Screening of Synthetic Molecules and Dietary Phytochemicals

**DOI:** 10.1371/journal.pone.0136509

**Published:** 2015-09-01

**Authors:** Hafsa Amat-ur-Rasool, Mehboob Ahmed

**Affiliations:** 1 Department of Microbiology and Molecular Genetics, University of the Punjab, Lahore, Pakistan; 2 National Center of Excellence in Molecular Biology, University of the Punjab, Lahore, Pakistan; University of Michigan, UNITED STATES

## Abstract

Alzheimer's disease (AD), a big cause of memory loss, is a progressive neurodegenerative disorder. The disease leads to irreversible loss of neurons that result in reduced level of acetylcholine neurotransmitter (ACh). The reduction of ACh level impairs brain functioning. One aspect of AD therapy is to maintain ACh level up to a safe limit, by blocking acetylcholinesterase (AChE), an enzyme that is naturally responsible for its degradation. This research presents an *in-silico* screening and designing of hAChE inhibitors as potential anti-Alzheimer drugs. Molecular docking results of the database retrieved (synthetic chemicals and dietary phytochemicals) and self-drawn ligands were compared with Food and Drug Administration (FDA) approved drugs against AD as controls. Furthermore, computational ADME studies were performed on the hits to assess their safety. Human AChE was found to be most approptiate target site as compared to commonly used Torpedo AChE. Among the tested dietry phytochemicals, berberastine, berberine, yohimbine, sanguinarine, elemol and naringenin are the worth mentioning phytochemicals as potential anti-Alzheimer drugs The synthetic leads were mostly dual binding site inhibitors with two binding subunits linked by a carbon chain i.e. second generation AD drugs. Fifteen new heterodimers were designed that were computationally more efficient inhibitors than previously reported compounds. Using computational methods, compounds present in online chemical databases can be screened to design more efficient and safer drugs against cognitive symptoms of AD.

## Introduction

Alzheimer's disease (AD), the most common cause of dementia in older people, is a progressive neurodegenerative disorder [1]. In year 2013, globally 44 million people suffered from this disease and the figure is predicted to rise up to 135 million sufferers by 2050 [2]. The disease is associated with appearance of plaques and tangles in brain tissue that gradually kills neurons in brain cortex, hippocampus, amygdala and certain other brain regions [3]. Due to non-regenerateable nature of neurons, the levels of acetylcholine (ACh) neurotransmitter produced by them declines [1]. This is known as cholinergic-deficit hypothesis for the AD [4]. Normally ACh is broken down instantly, as soon as it is produced, due to the activity of enzyme known as acetylcholinesterase (AChE) present in neural synapse [5]. Sufficient ACh is required for proper brain functioning, but due to decreased ACh in AD patients, they suffer from progressive decline in cognitive functioning (thinking, remembering, and reasoning) and behavioral abilities [6]. The symptoms start usually at age of 65 years with short term memory loss, lately long term memory loss and eventually lead to death due to multiple organ failure within approximately 8 years of onset [7, 8]. There is no effective treatment available till date, but inhibition of ACh breakdown by blocking the AChE, has been proved to be helpful in slowing down the disease progression [9].

Nearly all FDA approved drugs for AD therapy are acetylcholinesterase inhibitors [10] and their effectiveness is credited to the degree of inhibition of the enzyme [11]. Tacrine approved by FDA in 1993 was the first AChE inhibitor used for the treatment of AD [12]. Currently only few other AChE inhibitors such as donepezil [13], galantamine [14], huperzine-A [15] and rivastigmine [16] are generally used for treating cognitive symptoms in persons with mild or moderate AD [11]. But there are certain adverse effects associated with these inhibitors like dizziness, headache, constipation, hepatotoxicity, nausea, diarrhea, and bioavailability complications [17]. This stimulated the researchers to discover AChE inhibitors that are more effective and preferably produced by natural resources in order to minimize the side effects of the drugs [18]. Various experimental and computational approaches are in use for bioavailability studies, as a result compounds with poorer physical and chemical properties are filtered out at earlier stages before they enter clinical trials [19]. Until now about 400 inhibitors of AChE have been reported as alternate treatment options for AD [20].

Acetylcholinesterase is naturally abundant in electricity generating organs of torpedo ray and the X-ray crystal structures for torpedo AChE (tAChE) has been determined long before [21]. Since then, tAChE has been an extensively used source of the enzyme in AD therapy research [22]. Many reported synthetic and natural AD drug candidates as well as FDA approved drugs had been analyzed by using tAChE complexes instead of hAChE [13–15, 23]. Later on, Cheung et al. [24] reported that donepezil binds to tAChE in a significantly altered conformation as compared to recombinant hAChE (PDB: 4EY7) indicating that hAChE is more specific for drug discovery research [24].

In the present work, a comparison of human and torpedo AChE enzymes was done at sequence and structural levels to check dissimilarities. Docking studies were performed by using modeled hAChE to get more specific results for the human enzyme. Derivatives and heterodimers of FDA approved drugs for AD as well as phytochemicals with anti-AChE activity, retrieved from online databases, were screened on the basis of docking score and ADME criteria. Moreover, on the basis of information obtained from lead structures, we designed more efficient heterodimer leads that can be further be tested experimentally to become second generation drug candidates for AD.

## Tools and Methods

### hAChE Vs. tAChE

As no required reference amino acid sequences of hAChE Vs. tAChE were available in databases, representative amino acid sequences of hAChE (PDB: 2X8B chain A) and tAChE (PDB: 1EVE) were selected on the basis of multiple sequence alignment using Clustal Omega (http://www.ebi.ac.uk/Tools/msa/clustalo/). These sequences were pairwise aligned by using EMBOSS-Needle (http://www.ebi.ac.uk/Tools/psa/emboss_needle/). Pairwise alignment of 3D protein structures was performed between PDB entry 2X8B chain A for human and PDB entry 1EVE for *Torpedo californica* AChE by using online tool ALADYN (http://aladyn.escience-lab.org/) [25].

### Homology Modeling of hAChE

The amino acid sequence GI: 295321523 (PDB: 2X8B_A) comprising of 583 amino acid residues was retrieved from NCBI-protein database and homology modeled by using Phyre2 V 2.0 server (http://www.sbg.bio.ic.ac.uk/phyre2/html/page.cgi?id=index) [26]. The server uses PSI-BLAST to find homologue templates to model the 3D structure of provided sequence accordingly [27]. The predicted structure was pairwise aligned with PDB entries 2X8B_A and 4EY4 by using FATCAT server (http://fatcat.burnham.org/fatcat-cgi/cgi/fatcat.pl?-func=pairwise) [28].

### Molecular Docking

FDA approved drugs for AD (controls), their derivatives and heterodimers, food, Generally Recognized As Safe (GRAS) and medicinal grade phytochemicals with anti-AChE activity according to Dr. Duke's phytochemical and ethnobotanical databases (http://www.ars-grin.gov/duke/) [29] were retrieved from three different databases (NCBI-PubChem Compound, ChemSpider and DrugBank). The modeled hAChE was docked with 329 hits. PatchDock server (http://bioinfo3d.cs.tau.ac.il/PatchDock/)[30] was used as molecular-docking tool in order to determine enzyme (hAChE)-inhibitor binding affinities. The server was operated at Clustering RMSD value 4.0 and enzyme-inhibitor complex type. Results were evaluated by using top five poses for each hits on the basis of score, approximate interface area of the complex, atomic contact energy (ACE) and 3D transformation values. The output images were generated with PyMOL v 1.3 with hydrogen bonds lengths measured in Å.

The score values of FDA approved drugs were used as control, and were compared with that of hits by using following formula:

$$\%\;increase\;or\;decrease\;=\;\frac{(Score\;of\;hit–Score\;of\;control)\;\times \;100}{Score\;of\;control}$$

### *In-silico* ADME Prediction

Top scoring 40 hits and 10 phytochemical hits were subjected to computational ADME (absorption, distribution, metabolism, and excretion) studies by using online Lipinski filter supported by SCFBio (http://www.scfbio-iitd.res.in/software/utility/LipinskiFilters.jsp). The classical five properties for druglikeliness were analyzed i.e. molecular mass, octanol/water partition coefficient (CLogP), number of H-bond donors, number of H-bond acceptors and molecular refractivity.

### Heterodimer Designing

Fifteen new heterodimers were designed on the basis of molecular structure analysis of leads. The lead phytochemicals berberastine and berberine were joined with tacrine and pyrimidine with a C-linker by using ChemDraw Ultra 8.0 [31]. Docking studies were performed and their druglikeliness was determined by methods described above.

## Results and Discussion

### Comparison of tAChE and hAChE

**Fig 1:**
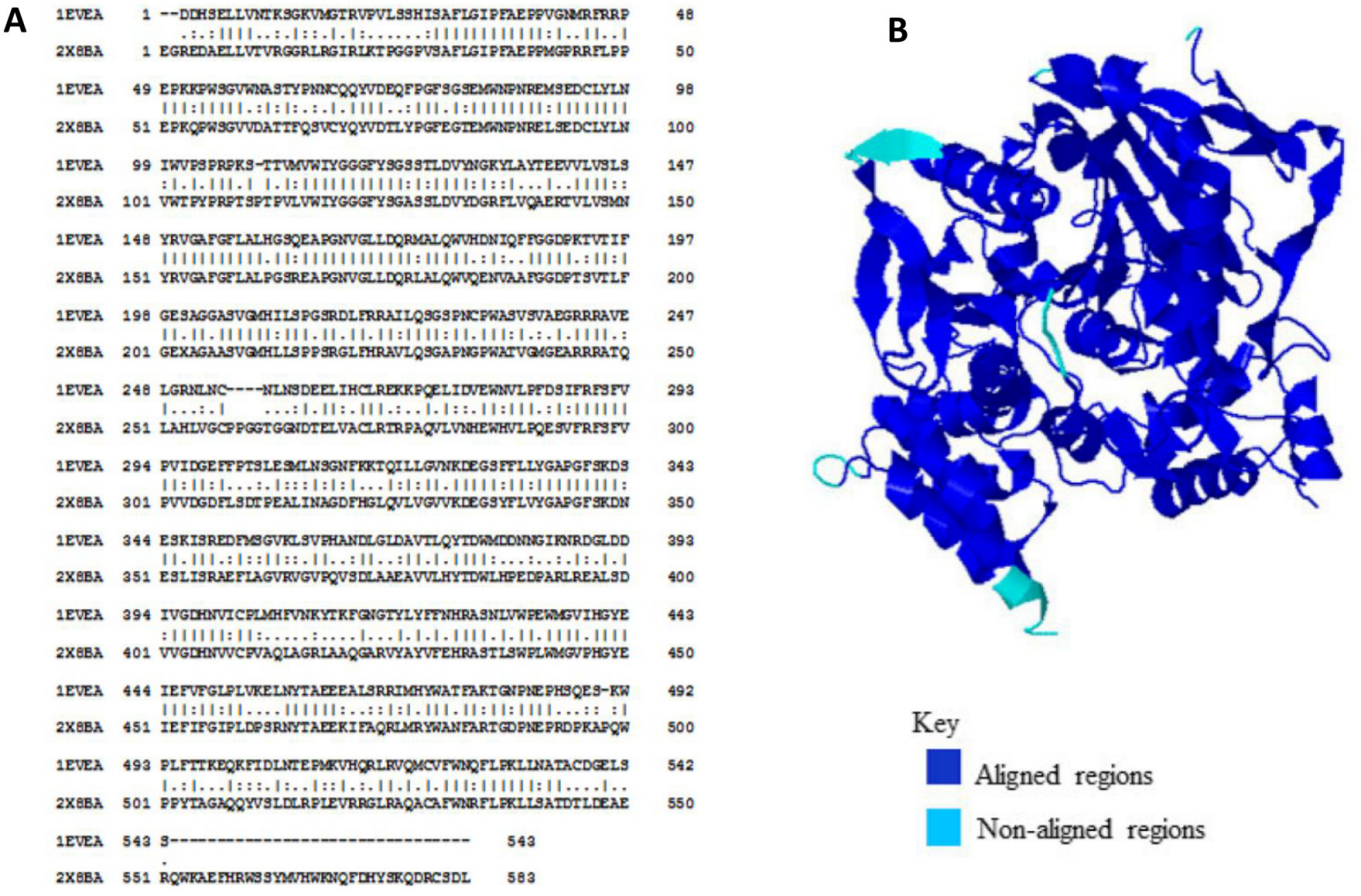
Alignment of human and *Torpedo californica* AChE. (A)Amino acid sequence alignment generated by RMBOSS-Needle. (B) 3D structure alignment of PDB entry 2X8B chain A for human and PDB entry 1EVE for *Torpedo californica* AChE generated by online tool Aladyn.

Pairwise alignment of human and *Torpedo californica* AChE sequences showed multiple mismatches and gaps between the two sequences. EMBOSS-Needle calculated 53.3% identity and 69.6% similarity between the two sequences using EBLOSUM62 matrix. The alignment generated is shown in Fig 1A. As the tertiary structures protein of remained more conserved in evolution than their amino acid sequences, alignment was performed between 3D structures of hAChE (2X8B_A: 536 a.a) and *Tc*AChE (1EVE: 534 a.a). A total of 517 amino acid residues were perfectly aligned between the two proteins and root mean square distance (RMSD) of the aligned regions was 1.8 Å. By using FATCAT algorithm [28] percentage similarity was calculated between the two structures that came out to be 75%. 3D alignment of the two structures is shown in Fig 1B. The difference between the human and torpedo enzyme at the levels of sequence and structure can probably have significant effect on ligand binding conformations.

### Homology Modeling of Human Acetylcholinesterase

**Fig 2:**
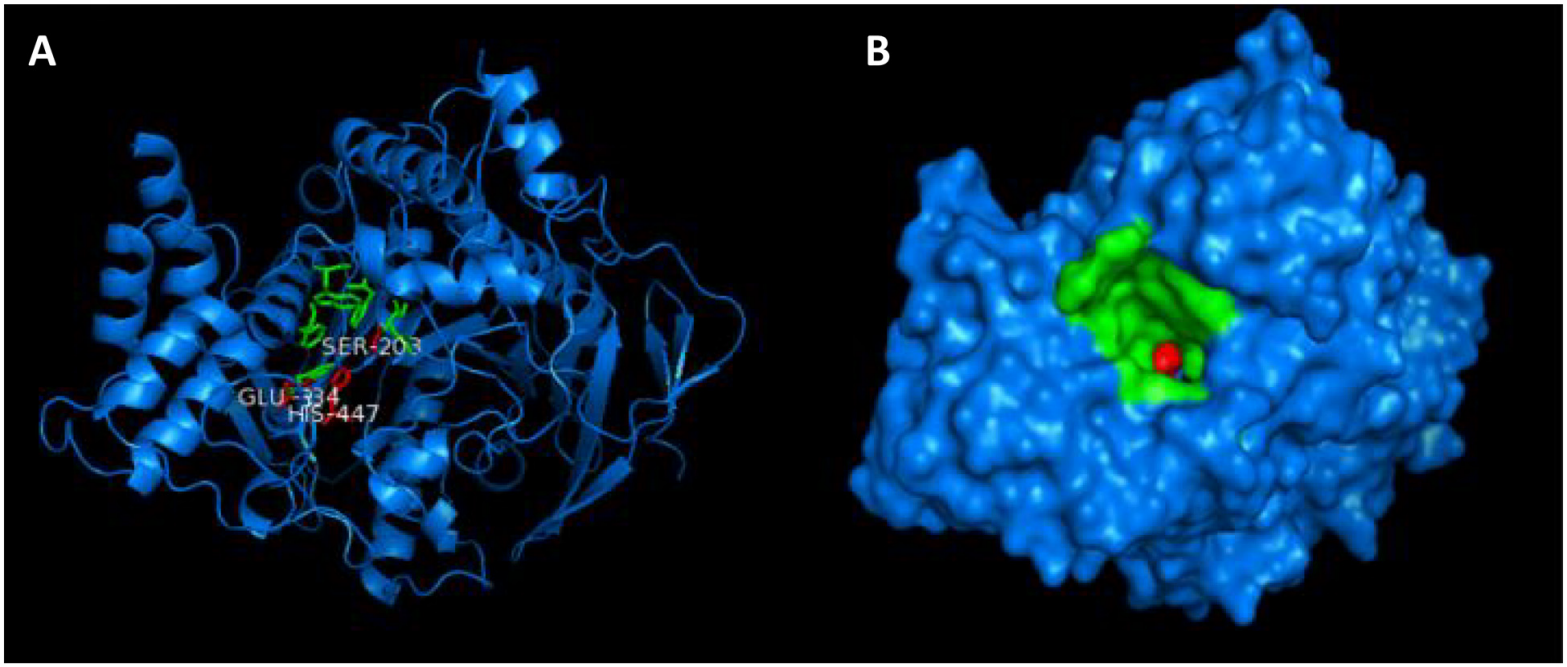
hAChE 3D structure modeled by Phyre2. (A) Cartoon representation, key residues are represented in green sticks with the catalytic triad in red. (B) Surface representation, looking down catalytic groove. Images generated by using PyMOL v1.3.

The Phyre2 (Protein Homology/AnalogY Recognition Engine) is one of most popular methods for protein structure prediction [26]. The server found maximum similarity of the target sequence provided (GI: 295321523) with fold library id: d2ha2a1. It used X-ray crystal structure d2ha2a1 as template and modeled the target. A total of 536 residues out of 583 residues (92% of sequence) were modeled with 100% confidence. The software classified the enzyme as superfamily alpha/beta-hydrolases and family as acetylcholinesterase-like enzyme. The modeled hAChE structure alignment with PDB entries 2X8B_A and 4EY4 (the two crystal structures available for human enzyme in PDB) by using FATCAT algorithm, showed 99.3% and 99% similarity with RMSD 0.32 and 0.65 respectively. The high percentage similarity indicates that the modeled hAChE (99% similar) is a better target for molecular docking as compared to torpedo enzyme (75% similar). Images of modeled hAChE indicating the active site is shown in Fig 2A and 2B.

### Molecular Docking and Druglikeliness

Five commonly used FDA approved drugs for AD (Donepezil, Galantamine, Huperzine A, Rivastigmine and Tacrine) were selected and docked with the hAChE. These all turn down the breakdown of acetylcholine in the brain. This lead to increased levels of acetyl-choline in the brain, and may preserve brain function [32, 33]. The docking score values came out to be in the order of: Donepezil (5998) > Rivastigmine (5998) > Galantamine (4676) > Huperzine A (4232) > Tacrine (4872). According to a Consumer Reports, when the efficiency of these treatments for AD were compared, majority of patients (upto 43%) left tacrine treatment due to its side effects, whereas this ratio was significantly lower for donepezil and galantamine treatment (19 and 17% respectively) [34]. So docking score can give us an information about the efficiency of possible drug. Out of synthetically designed database hits, CID: 21158810 came out to be the highest scoring synthetic compound fulfilling the criteria of ADME [19]. Present study showed that the hit CID: 21158810 is 81% more effective inhibitor as compared to tacrine and 19% more than that of donepezil (Table 1). Moreover, the Table 1 indicates that majority of the synthetic leads are dual binding site inhibitors i.e. having two binding subunits with a chain of usually 8–12 C atoms between individual components. These inhibitors bind to active site as well as catalytic groove of acetylcholinesterase and belong to second generation AD drugs category [35]. As they bind the target at two sites they are more potent inhibitors [36]. Lipinski's rule of five is traditionally used to evaluate druglikeness or oral bioavailability of drugs in humans. It identifies five critical properties that are molecular mass <500 Da, octanol/water partition coefficient (LogP) <5, number of hydrogen-bond donors <5, number of hydrogen-bond acceptors <10 and molecular reactivity between 40 and 130 [19]. The rule describes molecular properties important for ADME (absorption, distribution, metabolism, and excretion), but, the rule does not predict pharmacological activity. It predicts high probability of clinical failure for molecules disobeying two or more of the rules [19]. The criteria for molecular weight <500Da was modified to <600Da to increase the hit rate, as the big active site of the enzyme require bigger drug molecules to cover it (S1 Table) [37].

**Table 1:** Details of top leads fulfilling ADME criteria, and their score comparison to FDA approved drugs (Tacrine, Huperzine A, Galatamine, Rivastigmine, Donpezil) for AD.

Lead type	Ligand	2D Structures	Score	%age Increase/Decrease in docking Score as Compared to FDA approved drugs for AD				
				Tacrine	Huperzine A	Galatamine	Rivastigmine	Donpezil
SL-1	CID_21158810	Fig 3A	7120	81	68	52	46	19
SL-2	CID_5326967	Fig 3B	6934	76	64	48	42	16
SL-3	CID_23645226	Fig 3C	6872	74	62	47	41	15
SL-4	CID_16094871	Fig 3D	6726	71	59	44	38	12
SL-5	CID_5326966	Fig 3E	6640	69	57	42	36	11
PL-1	Berberastine	Fig 3F	5520	40	30	18	13	-8
PL-2	Berberine	Fig 3G	5462	39	29	17	12	-9
PL-3	Yohimbine	Fig 3H	5104	30	21	9	5	-15
PL-4	Sanguinarine	Fig 3I	4938	25	17	6	1	-18
PL-5	Elemol	Fig 3J	4338	10	3	-7	-11	-28
DHD-1	Berberastine-5C-Pyrimidine	Fig 3K	7510	91	77	61	54	25
DHD-2	Berberine-5C-Pyrimidine	Fig 3L	7428	89	76	59	52	24
DHD-3	Berberine-4C-Pyrimidine	Fig 3M	7334	86	73	57	51	22
DHD-4	Berberastine-5C- Tacrine Derivative	Fig 3N	6992	77	65	50	44	17
DHD-5	Berberastine-3C- Tacrine Derivative	Fig 3O	6726	71	59	44	38	12

SL, Synthetic lead; PL, Phytochemical lead; DHD, Designed heterodimer

**Fig 3:**
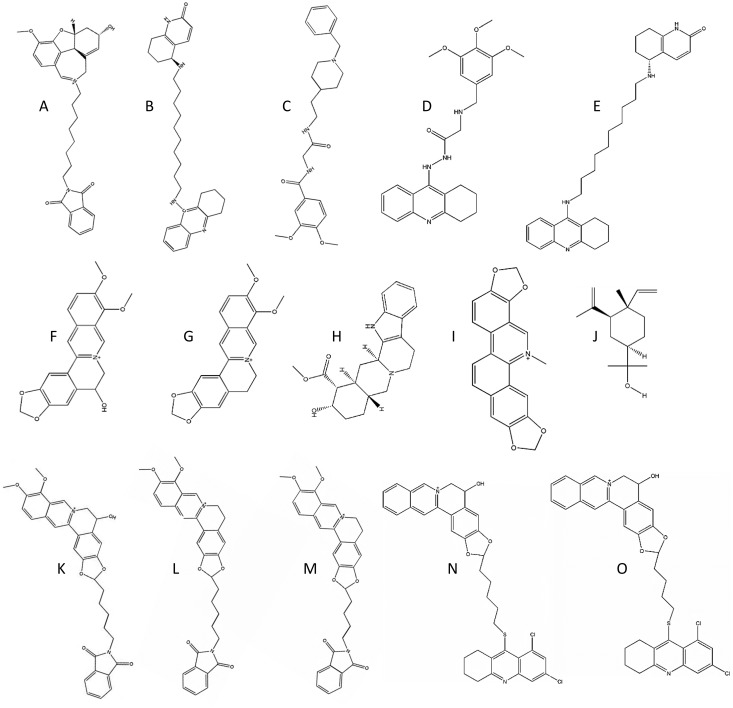
Two dimentional structures of top leads used in study. (A-E) Synthetic leads. (F-J) Phytochemical leads. (H-O) Designed heterodimers.

The set of medicines already in practice manifest severe side effects including nausea, vomiting, diarrhea, headache, insomnia and many other complications. Furthermore the treatment is very expensive, ranging in cost from $177 to more than $400 per month (PKR 18,000 to 40,000) [34]. By taking into account the side effects of chemical drugs, dietary phytochemicals were screened for a purpose to find edible plant sources as an alternate treatment of AD. The top most phytochemical showed 40% more efficient binding with receptor as compared to tacrine, however, none of them were as effective as donepezil (Table 1). Berberastine, berberine, yohimbine, sanguinarine, elemol and naringenin are the worth mentioning phytochemicals in this context. The concentrations (ppm) of these phytochemicals in major source plants are given in Table 2. *Hydrastis canadensis* (common name ‘goldenseal’), containing berberastine and berberine, is a multi-purpose homeopathic remedy [38]. *Coptis chinensis* (Chinese goldthread) is one of the 50 basic herbs used in traditional Chinese medicine and its rhizome is a source of alkaloids like berberine and others [39]. *Berberis vulgaris* (barberry), a shrub that produces edible berries rich in vitamin C, is a good source of berbrine [40]. Ji and Shen [41] reviewed anti-AChE activity of berberine to combat Alzheimer’s disease. They reported the theoretically estimated binding affinities of berberine to the enzyme AChEare very close to the experimental values. *Catharanthus lanceus* (known as ‘sadabahar’ in South Asia) is medicinal plant and contains 6% yohimbine in its leaves [42]. Mroue and coworkers showed anti-AChE activity of β-yohimbine with IC_50_ 0.431mMin vitro [43]. *Sanguinaria canadensis* (blood root) is a historically used medicinal plant by Native Americans [44]. The plant contains sanguinarine and according to Houghton et al. [45] this phytochemical showed in vitro activity of IC_50_ 0.23 μM against bovine erythrocyte AChE. *Citrus sinensis* (sweet orange) contain elemol and naringenin that also showed significant anti-hAChE activity in present study. Heo et al. [46] found out that when naringenin was administered at a concentration of 4.5 mg/kg body weight of mice, it significantly reduced induced forgetfulness.

**Table 2:** Occurrence of the phytochemical leads in nature [29].

Phytochemical	Source Plant	Concentration (ppm)
Berberastine	*Hydrastis canadensis*	60000
Berberine	*Coptis chinensis*	180000
	*Coptis japonica*	140000
	*Hydrastis canadensis*	120000
	*Berberis vulgaris*	60000
Yohimbine	*Catharanthus lanceus*	100000
Sanguinarine	*Sanguinaria canadensis*	120000
Elemol	*Citrus sinensis*	56400
Naringenin	*Citrus sinensis*	91600

**Fig 4:**
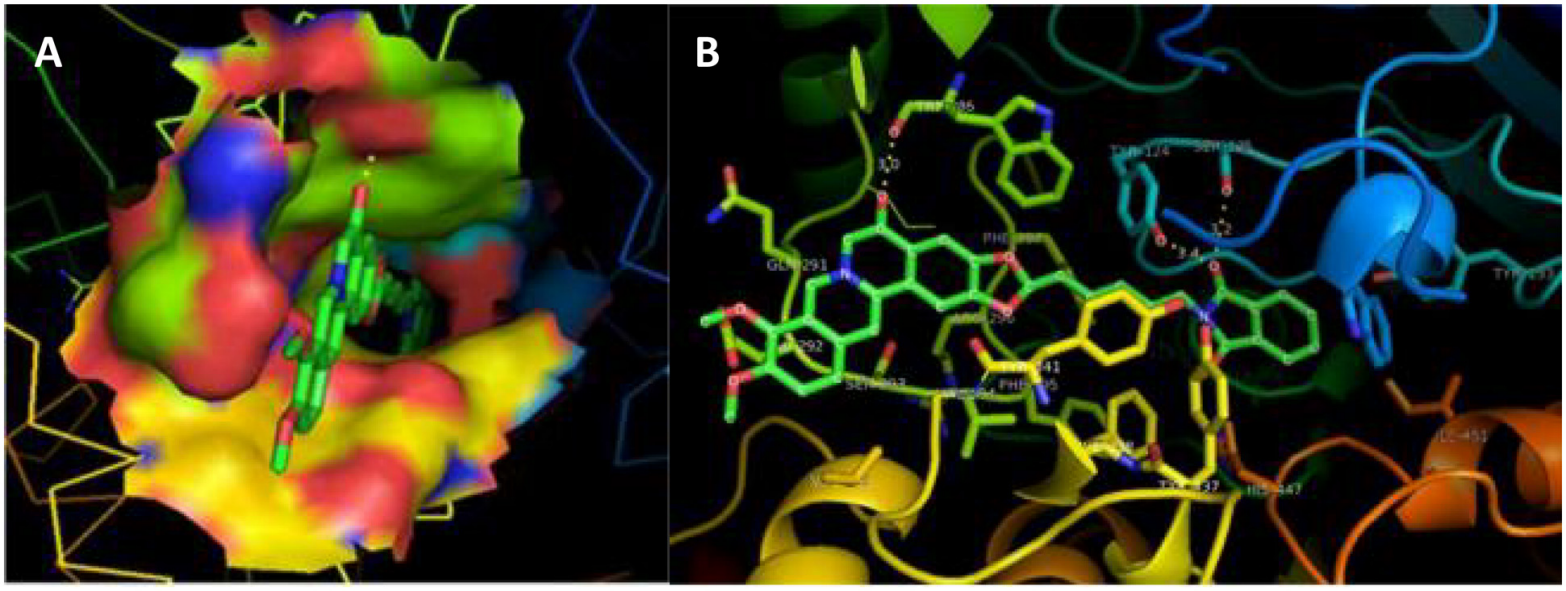
Visual results of docking of best designed AD drug candidate, berberastine-5C-pyrimidine, into modeled hAChE. (A) Ligand attachment conformation along the active site groove shown by solid surface ligand site. (B) Ligand attached with different residues of catalytic site, represented by sticks and colored according to the element type and element name is labeled. Active site residues are represented by sticks and enzyme by cartoons. Hydrogen bonding shown by yellow dashes and distance measured in Å.

One step forward, by considering the novel “multi-target-directed strategy” i.e. designing a molecule with dual or multiple binding sites [47], new heterodimers were planned. On the basis of structural analysis of synthetic and phytochemical leads (Table 1) new heterodimers were designed as combinations of natural alkaloids berberastine and berberine with tacrine and pyrimidine rings that are active components of top synthetic leads. These heterodimers can be potential second generation AD drugs in future. As inhibitors of the human enzyme instead of torpedo they are more specific in their activity, and fulfillment of druglikeliness criteria makes them orally safer. The 2D structure of the top five newly designed drugs along with their score comparison to controls is shown in Table 1. Present study demonstrates that, the designed lead berberastine-5C-pyrimidine is 91% more potent than tacrine, 25% more potent than donepezil and 10% more potent than the best synthetic database lead. Visual results for berberastine-5C-pyrimidine docking with the modeled hAChE (Fig 4A) shows that the pyrimidine ring blocks the catalytic site, five carbon chain span along the length of catalytic groove and berberastine binds to the peripheral site. Fig 4B shows O-OH type hydrogen bonding of ligand with TRP-286, TYR-124 and SER-125 residues of hAChE. The distances of hydrogen bonds were kept at < 3.50 Å cutoff.

## Conclusion

The present study is aimed on finding more effective hAChE inhibitors to save acetylcholine neurotransmitter that in turn can slowdown AD progression. The comparison of human and torpedo AChE enzymes at protein sequence and structure level performed in present study showed much dissimilarity that indicates that hAChE is a better drug target then tAChE. We performed docking and druglikeliness studies to elucidate the effectiveness and biological safety of synthetic molecules and dietary phytochemicals present in online chemical databases as inhibitors of hAChE. By using lead compounds of database screening we designed more highly scoring leads that gave valuable results in further lab and clinical testing to give a better relief to the AD patients against cognitive symptoms.

## Supporting Information

S1 Table Lipinski’s filer values of tested lead compounds. (XLSX)
